# All for one: Collaboration between dermatologist, radiation oncologist and radiologist in the clinical management of “difficult to treat” non melanoma skin cancer

**DOI:** 10.1016/j.ctro.2024.100774

**Published:** 2024-03-30

**Authors:** Federico Gagliardi, Anna Russo, Camila Scharf, Alessandro Pinto, Mario Faenza, Emma D'Ippolito, Giuseppe Argenziano, Teresa Troiani, Alfonso Reginelli, Valerio Nardone

**Affiliations:** a Department of Precision Medicine, University of Campania “L. Vanvitelli”, Naples 80138, Italy; b Dematology Unit, University of Campania L.Vanvitelli, Naples, Italy; c Multidisciplinary Department of Medical, Surgical and Dental Sciences, Plastic Surgery Unit, Universityof Campania “Luigi Vanvitelli”, Naples, Italy

**Keywords:** non-melanoma skin cancer (NMSC), Radiotherapy, high-frequency ultrasound (HFUS), Dermascopy

## Abstract

This series introduces the clinical management of difficult-to-treat non-melanoma skin cancers (NMSCs) through a multidisciplinary approach, emphasizing the integration of dermoscopy and Ultra high-frequency ultrasound (UHFUS) for accurate diagnosis and treatment planning, particularly in cases referred for radiotherapy (RT). Dermoscopy aids in diagnosing both pigmented and non-pigmented skin lesions, guiding treatment margins and reducing the benign-to-malignant biopsy ratio. UHFUS provides valuable insights into tumor size, depth, and vascularity, complementing clinical evaluations and assisting in RT planning. Three challenging cases are presented, highlighting the pivotal role of dermoscopy and UHFUS in decision-making and treatment optimization. Collaboration between dermatologists, radiation oncologists, and radiologists enhances diagnostic accuracy, tailoring treatment plans to individual patient needs and preferences, ultimately improving patient outcomes and experience. The integration of these imaging techniques holds promise for optimizing non-surgical treatments like RT and monitoring treatment progress, offering a personalized approach to NMSC management.

## Highlights

• Multidisciplinary Approach: The article emphasizes the importance of a multidisciplinary approach in managing difficult-to-treat non-melanoma skin cancers (NMSCs), particularly those referred for radiotherapy (RT).
• Integration of Dermoscopy and HFUS: Dermoscopy and high-frequency ultrasound (HFUS) are highlighted as essential diagnostic tools that complement clinical evaluations and assist in treatment planning for NMSCs.
• Treatment Optimization: The article presents three challenging cases, demonstrating how the integration of dermoscopy and HFUS contributes to treatment optimization, particularly in cases of recurrence or complex lesions.

## Introduction

Skin cancer like basal cell carcinoma (BCC) and squamous cell carcinoma (SCC), along with melanoma, predominantly affects individuals with fair complexions due to long-term exposure to ultraviolet (UV) radiation [1]. Early detection and treatment are crucial, typically involving surgical excision, Mohs surgery, radiation therapy, or topical medications, chosen based on tumor characteristics [2]. Some cases, labeled as 'difficult-to-treat', pose challenges due to factors like tumor size, location, and patient-related issues. In such cases, noninvasive imaging techniques like dermoscopy and UltraHigh-Frequency Ultrasound (UHFUS) aid in diagnosis, margin assessment, and treatment planning [3].

Dermoscopy, utilizing magnification devices and liquid application, enables visualization of skin lesions in the epidermis and upper dermis, enhancing diagnostic accuracy for pigmented and non-pigmented lesions. Despite requiring significant clinician experience, it reduces unnecessary biopsies and facilitates earlier diagnosis, with ongoing advancements promising further enhancements in diagnostic capabilities [4].

High-frequency ultrasound (HFUS) up to 22 MHz has been used for measurement of skin thickness in patients in various clinical contexts; instead UHFUS represents a new imaging approach for diagnosing and guiding microsurgery, particularly in anatomical structures that are difficult to assess using conventional HFUS methods.[5].

In facts, UHFUS, operating at 20–100 MHz, provides high-resolution images for assessing NMSCs, including margin evaluation and vascularization assessment. Despite limited penetration depth, it offers superior spatial resolution compared to HFUS, aiding in early detection and accurate assessment of skin lesions [6], [7], [8].

Here we present a series of cases of locally advanced ‘difficult-to-treat’ skin cancer that were evaluated in a multidisciplinary setting, with the help of dermoscopic examination by the dermatologist, UHFUS by the radiologist and clinical assessment by the radiation oncologist for deciding the most appropriate approach.

## Methods

In Multidisciplinary Tumor Board (MTB) meetings, all non-melanoma skin cancer (NMSC) cases are thoroughly reviewed. For challenging cases referred for radiotherapy, such as those with difficult localizations or unclear margins, a joint consultation is initiated by the radiation oncologist, involving a dermatologist, a radiation oncologist, and a radiologist. This collaborative approach ensures comprehensive evaluation and management.

Dermoscopic evaluation utilized a Fotofinder Handyscope dermatoscope (10x magnification) to assess macroscopic features, surface characteristics, vascularization, and clinical margins.

Ultrasound scans were conducted by an experienced radiologist with a decade of ultrasound expertise with 5 years of high frequencies experience, using a Vevo ultrasound machine (FUJIFILM VisualSonics).

UltraHigh-Frequency Ultrasound (UHFUS) provided detailed lesion assessment, especially in areas where clinical inspection and dermoscopy were insufficient. It aided in visualizing invisible margins and delineating the Gross Tumor Volume (GTV).

Radiotherapy (RT), when deemed suitable, was administered using electron or photon beams based on tumor depth estimation. The GTV included the lesion area identified by dermoscopy and UHFUS plus a 1.5 cm margin for microscopic extension to create the planning target volume (PTV). RT was delivered with an Elekta Linear Accelerator.

Three cases of difficult-to-treat NMSC managed with dermatoscopic and UHFUS evaluation are presented. All procedures followed the principles of the Declaration of Helsinki (1964).

## Case descriptions

### Clinical case 1

Since 2016, the patient has been known in dermatology due to a diagnosis of several basal cell carcinomas (BCC) and has a history of kidney cancer, resulting in a single functioning kidney and dialysis since 2020. Due to multiple tumor locations, the patient initiated vismodegib therapy in 2016, which continued until 2019 alongside localized surgical excisions. Despite experiencing side effects such as hair loss, weight loss, and muscle spasms, the vismodegib was well tolerated until it was discontinued due to disease progression in the auricular region and an increase in creatinine levels. In 2020, surgical excision of the retroauricular lesion was performed, followed by adjuvant radiotherapy with a total dose of 50 Gy in 25 fractions. However, in 2022, a local recurrence in the retroauricular region led to treatment with sonidegib. Due to further local disease progression, the patient was subsequently referred to the radiotherapy department for clinical evaluation.

Clinically the margins of the BCC were not clearly visible but, under dermoscopy, all the area of recurrence on the pre-auricular region was observed with ovoid nests and arboriform telangiectasias.

The retroauricular area did not show any signs of recurrence.

**Fig. 1:**
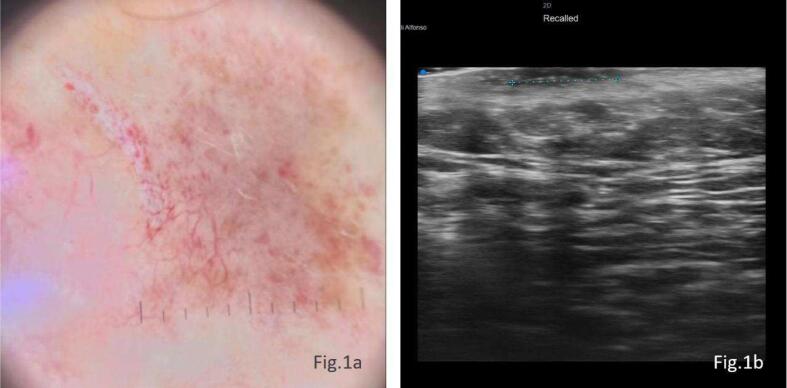
The image shows a BCC lesion with irregular borders, ovoid nests, and arboriform telangiectasias (Fig. 1a); UHFUS images show a lesion characterized by subepidermal growth and an oval morphology with indistinct borders and a hypoechogenic echostructure (Fig. 1b).

At the pre-auricular level, UHFUS shows a lesion with subepidermal development and oval morphology. The lesion has poorly defined borders and a shaded hypoechogenic ecostructure with a maximum size of approximately 0.92 × 5.3 mm, which showed an intense signal on ColorDoppler. This finding suggests disease recurrence (Fig. 1a-b).

Clinical Decision: the patient chose not to undergo radiotherapy due to logistical challenges, particularly as a dialysis patient, making it difficult to adhere to scheduled treatment appointments at the facility. This decision emphasizes the importance of considering individual patient circumstances in treatment planning. Currently, the patient remains on treatment with sonidegib and is closely monitored, with plans for a salvage radiotherapy approach when necessary.

### Clinical case 2

A 74-year-old patient, who previously underwent a liver transplant and is currently on everolimus therapy, was diagnosed with a squamous cell carcinoma (SCC) of the scalp, Grade 3, in 2019. Surgical excision was performed followed by adjuvant radiotherapy at another center, delivering a total dose of 70 Gy in 35 fractions. Since 2022, the patient has developed multiple squamous cell skin carcinomas on the scalp, necessitating local excisions. However, these procedures resulted in a large ulcerated lesion with exposed bone (refer to the figure). Due to local disease recurrence, with an ulcerated and bleeding lesion found in the previously treated area, the patient’s case was reviewed for consideration of reirradiation.

**Fig. 2:**
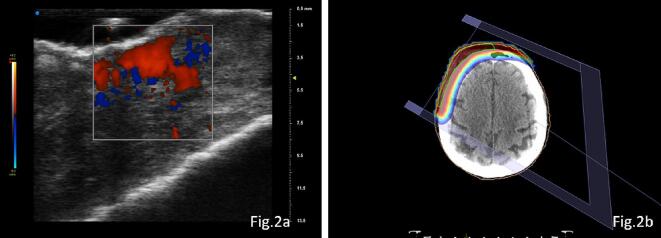
The UHFUS images reveal irregular margins with increased echogenicity on the epidermal side and a diffusely heterogeneous and hypoechogenic echostructure. A pronounced vascular signal is evident on colour Doppler imaging (Fig. 2a); RT treatment planning simulates the direction of the electron beam and the distribution of isodoses.This visual representation allows for precise planning, ensuring accurate targeting of the marked target area with HFUS (Fig. 2b).

Dermatologic visit indicated local recurrence on the inferior and left superolateral margin, with white circles and polymorphic vessels under dermoscopy. The UHFUS shows an ulcerated lesion with irregular and hyperechogenic margins on the epidermal side with a subepidermal depth of approximately 4 mm. The ecostructure appears diffusely inhomogeneous and hypoechogenic. Additionally, there is an increased vascular signal on ColorDoppler (Fig. 2a). The lesion in the ulcerated tract is indicative of disease recurrence. Incisional biopsy confirmed SCC, and it was decided to manage the recurrence with re-irradiation. The synergy between the two imaging techniques were pivotal to reduce the field of reirradiation in this complex case.

*Clinical decision:* The patient was treated with radiotherapy, with a dose of 36 Gy in 9 fx (due to the previous high dose exposure). The combined use of both imaging techniques played a crucial role in minimizing the reirradiation field in this intricate case (Fig. 2b). At the end of the treatment, the patient reported grade 1 erythema and oedema, according to the Common Terminology Criteria for Adverse Events v.5.0 (CTCAE) scale. The patient is still in follow-up, with no signs of recurrence of disease.

### Clinical case 3

The patient of 88 years old with a ullous lesion of the scalp that was treated with a surgical excision.

The pathological exam had the morphological finding of a neoplasm deriving from the follicular appendages, with matric differentiation, with compatible characteristics (in consideration of the presence of necrosis and atypical mitoses) with pilomatric carcinoma, with a focal distance from the margins less than 1 mm. The patient was referred to our Department to consider an adjuvant radiotherapy.

At the left fronto-parietal level, under dermoscopy, post operative telangiectasias and signs of photodamaged skin could be seen but no clear sign of recurrence was evident. On the left temporal region, however, a millimetric ulcerated plaque, was observed, with peripheric white circles under dermoscopy.

**Fig. 3:**
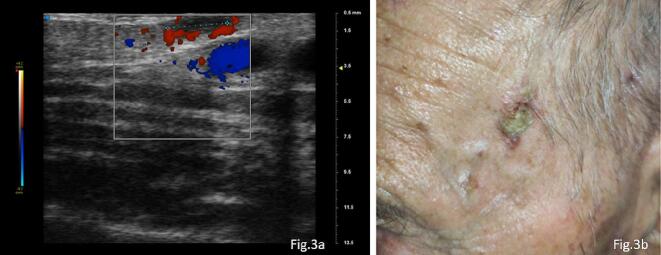
The UHFUS examination showed an oval shape with well-defined margins and a hypoechogenic structure with a thin signal on colour Doppler imaging (Fig. 3a). At the last follow-up, the lesion had grown significantly since the previous visit, it was excised and identified as SCC (Fig. 3b).

UHFUS scan shows an oval lesion with regular margins, and a hypoechogenic ecostructure. Its maximum diameter is about 3.35 mm x 1.20 mm. The lesion showed intense signal on ColorDoppler and is compatible with actinic keratosis. UHFUS of the left temporal lesion had an oval shape, regular margins, and a hypoechogenic structure. Its maximum dimensions were about 2.86 mm x 0.96 mm, and it had a modest signal at ColorDoppler. This lesion exhibited signs of malignancy (Fig. 3a).

*Clinical decision:* Despite the detection of a small suspicious recurrence of the disease, the patient and his relatives decided to undergo close monitoring, taking into account his age and the low perceived risk. This decision is based on the patient’s specific situation and is in line with a personalized and conservative management strategy. Given the heightened suspicion of malignancy during the subsequent follow-up examination, the patient underwent a surgical excision of the left temporal lesion, revealing an in situ squamous cell carcinoma (Fig. 3b).

## Discussion

The Multidisciplinary Tumor Board (MTB) is crucial for cancer management, with studies showing improved survival rates in patients with a history of multidisciplinary meetings. In managing “difficult-to-treat” non-melanoma skin cancers (NMSCs), comprehensive evaluation in a multidisciplinary setting is essential. Dermoscopy and high-frequency ultrasound (HFUS) are fundamental diagnostic tools utilized [9].

Dermoscopy aids in diagnosing NMSCs by identifying specific features such as vascular morphology, guiding treatment margins for radiotherapy. It plays a crucial role in delineating accurate tumor margins, necessary for both presurgical evaluation and radiotherapy planning [10]. However, its application in delineating lateral margins prior to radiotherapy remains limited [11], [12]. Collaboration between dermatologists and radiation oncologists optimizes treatment planning, reducing recurrence risks and minimizing healthy tissue damage [11], [12].

HFUS can be a useful tool for diagnosing skin lesions, with a sensitivity of at least 83 % for detecting melanoma. However, its specificity can vary, ranging from 33 % to 73 %.[13].

However, it is important to consider that defining lesion boundaries can be difficult in the presence of inflammation and scar tissue. Additionally, hyperkeratosis in some SCCs may obstruct imaging during ultrasound examinations. It may be helpful to remove scabs prior to examination [14].

HFUS provides insights into tumor size and depth, aiding in treatment decisions and monitoring, [6].

Studies indicate superior outcomes with HFUS-guided radiotherapy compared to non-imaging-guided approaches for NMSCs [15], [16], [17]. HFUS also shows promising results in achieving local control and low toxicity scores in NMSC treatment [17], [18].

## Conclusions

In future, emerging non-invasive diagnostic modalities may further improve treatment monitoring. Integrating dermoscopy and UHFUS enhances the clinical management of difficult-to-treat NMSCs referred for radiotherapy, emphasizing the importance of individualized treatment plans tailored to each patient's circumstances and preferences. Collaboration among dermatologists, radiotherapists, and radiologists streamlines the process, ensuring faster interventions and better patient experiences.

## CRediT authorship contribution statement

**Federico Gagliardi:** Investigation, Writing – original draft, Supervision. **Anna Russo:** Investigation, Writing – original draft. **Camila Scharf:** Investigation, Writing – original draft. **Alessandro Pinto:** Investigation, Writing – original draft. **Mario Faenza:** Investigation, Methodology. **Emma D'Ippolito:** Investigation, Writing – original draft, Methodology. **Giuseppe Argenziano:** Supervision, Investigation. **Teresa Troiani:** Supervision, Investigation. **Alfonso Reginelli:** Writing – review & editing, Supervision, Investigation. **Valerio Nardone:** Conceptualization, Supervision, Writing – review & editing, Investigation.

## Declaration of competing interest

The authors declare that they have no known competing financial interests or personal relationships that could have appeared to influence the work reported in this paper.
